# Variability, asymmetry and sexual dimorphism in craniofacial anomalies in Loeys-Dietz syndrome 2: geometric morphometric analysis in mice

**DOI:** 10.1038/s41598-026-35325-8

**Published:** 2026-01-10

**Authors:** Katelin R. Devine, Sarah Lynn, Priyam Jani, Cyrus Keyvanfar, Bikash Lamichhane, Ashleigh S. Hanner, Catharine Dietrich, Rachel S. Chung, Pamela A. Frischmeyer-Guerrerio, Konstantinia Almpani, Olivier Duverger, Janice S. Lee

**Affiliations:** 1 Craniofacial Anomalies and Regeneration Section, National Institute of Dental and Craniofacial Research, National Institutes of Health, 30 Convent Drive, Building 30, Room 202, Bethesda, MD 20892 USA; 2 Food Allergy Research Section, National Institute of Allergy and Infectious Diseases, National Institutes of Health, Bethesda, MD 20892 USA

**Keywords:** Loey-Dietz syndrome, Craniofacial anomalies, Geometric morphometric analysis, Variability, Asymmetry, Sexual dimorphism, Anatomy, Diseases, Genetics, Medical research

## Abstract

Loeys-Dietz syndrome is a rare connective tissue disorder characterized by life-threatening aortic aneurysm and distinctive craniofacial anomalies. It is caused by mutations along the transforming growth factor beta (TGF-β) signaling pathway (LDS1-6). We previously showed that craniofacial anomalies varied among LDS subtypes and that LDS2, caused by mutations in the *TGFBR2* gene, exhibited the most severe and variable phenotype. In this study, we performed a thorough qualitative and quantitative analysis of craniofacial anomalies in a mouse model for LDS2, through micro computed tomography and 3D geometric morphometric analysis at multiple postnatal stages. We show that craniofacial shape in *Tgfbr2*^*G357W/*+^ mice strongly deviates from their WT littermates from an early age and exhibit high variability and evidence of left–right asymmetry despite the pure genetic background. Cranial doming, shortening of the anterior part of the skull, widening of the space between orbits, reduction of mandibular size, suture fusion in the cranial vault and palate, and abnormal condylar shape were among features that were consistent with the phenotype seen in patients with LDS. Interestingly, several of these features were more prevalent and severe in females than in males, indicating potential sexual dimorphism, further supported by the trend observed in our revisited clinical data.

**Supplementary Information:**

The online version contains supplementary material available at 10.1038/s41598-026-35325-8.

## Introduction

Loeys-Dietz Syndrome (LDS) is a rare autosomal dominant connective tissue disorder exhibiting craniofacial anomalies in combination with life-threatening aortic aneurysms with high risk of rupture. It is caused by pathogenic variants in genes along the transforming growth factor-beta (TGF-β) signaling pathway^1,2^. There are six types of LDS that are defined by the gene affected. LDS mutations can be found in the genes encoding TGF-β receptor 1 (*TGFBR1*), TGF-β receptor 2 (*TGFBR2*), suppressor of mothers against decapentaplegic 3 (*SMAD3*), TGF-β2 ligand (*TGFB2*), TGF-β3 ligand (*TGFB3*), and suppressor of mothers against decapentaplegic 2 (*SMAD2*), which result in LDS Type 1 (LDS1, MIM #609,192)^1,3^, LDS Type 2 (LDS2, MIM #610,168)^1,3^, LDS Type 3 (LDS3, MIM #603,795)^4^, LDS Type 4 (LDS4, MIM #614,816)^5^, LDS Type 5 (LDS5, MIM #615,582)^6^ and LDS Type 6 (LDS6, MIM #619,656)^7^, respectively.

Although the classical triad of LDS is described as arterial tortuosity and aneurysm, hypertelorism, and bifid uvula or cleft palate, other craniofacial manifestations such as craniosynostosis and mandibular hypoplasia and/or retrognathism have also been associated with LDS^1,2^. Moreover, through thorough craniofacial evaluation of a cohort (n = 40) of patients with LDS using standard two-dimensional (2D) cephalometric analysis as well as three-dimensional (3D) geometric morphometric analysis of soft (3D photos) and skeletal (CBCT, cone beam computed tomography) tissues, we recently provided a more extensive description and quantitative evaluation of the craniofacial anomalies associated with the disease^1,8^. Oro-dental evaluation was also performed using clinical evaluation and intraoral photos^9^. These studies indicated that the craniofacial phenotype was highly variable in LDS with mandibular hypoplasia and/or retrognathism and flat midface projection being the most common features detected (> 80%). Interestingly, hypertelorism was seen in less than 50% of the patients. Moreover, although a high arched palate was commonly seen (> 80%) in patients with LDS, bifid uvula and submucous cleft palate were only observed in some patients with LDS1 or LDS2, and none of the patients exhibited a full cleft palate deformity. Other intraoral characteristics included dental malocclusion, dental crowding and delayed eruption of permanent teeth. The prevalence of all craniofacial and oro-dental features varied between LDS subtypes, with the highest severity and variability seen in patients with LDS2^8,9^. We further demonstrated poor oral health-related quality of life in patients with LDS, in part due to dental malocclusion and temporomandibular joint abnormalities^10^. Therefore, the craniofacial and oro-dental manifestations in patients with LDS present a challenge for the dental practitioners who treat them^11^.

The importance of TGF-β signaling in craniofacial development is well documented and various mouse models in which TGF-β signaling is disrupted during the development of craniofacial bones have been analyzed^12–15^. Targeted deletion of *Tgfbr2* in the cranial neural crest resulted in complete cleft palate, missing frontal bone, and shortening of the maxilla and mandible^12,13^. These phenotypes resulted in perinatal mortality, which precluded the analysis of postnatal craniofacial anomalies. Targeted deletion of *Tgfbr2* in osteoblast-lineage cells resulted in underdevelopment of the mandibles and the anterior part of the skull^15^, a phenotype that could be analyzed at postnatal stages and was consistent in all mutant mice. These mice died around four weeks after birth potentially due to craniofacial anomalies that affect breathing and feeding^16^.

Because LDS2 is an autosomal dominant disorder, patients with the disease have one copy of the mutant receptor and one copy of the wild-type receptor. So far, there has been no in-depth study looking at craniofacial anomalies caused by a heterozygous mutation in the *Tgfbr2* gene in mice. In the present study, we used a knock-in mouse model harboring a mutation that causes LDS2 in humans^1,3^ (*Tgfbr2*^*G357*^^*/*+^) to thoroughly characterize the craniofacial anomalies caused by heterozygous mutation in the *Tgfbr2* gene in mice. This inbred mouse model (pure genetic background) was previously shown to recapitulate the cardiovascular phenotype of LDS patients^17^, as well as the skeletal^18^, dental^19^, and gastrointestinal^20^ manifestations of the disorder. We therefore hypothesized that this mouse model also recapitulates the craniofacial anomalies observed in patients with LDS2 and may provide novel insights on their development. Craniofacial characterization was performed using qualitative as well as quantitative assessments, including 3D geometric morphometric analysis on a large cohort of mice (n = 84), at four different postnatal stages to appreciate the extend and progression of the morphological changes and identify characteristic craniofacial features caused by mutation in *Tgfbr2* in mice. This approach allowed us to (1) show that the animal model recapitulates the variability in craniofacial anomalies observed in patients with LDS2 starting at an early age, (2) determine that cranial asymmetry is a highly prevalent characteristic in this model, (3) identify characteristic craniofacial anomalies that are relevant to multiple aspects of the clinical manifestations in LDS2, and (4) reveal the element of sexual dimorphism in craniofacial anomalies in LDS2.

## Results

### Significant differences in cranial and mandibular shape with high variability are observed from an early age in ***Tgfbr2***^***G357W/***+^ mice

Skulls from *Tgfbr2*^*G357W/*+^ mice and wild-type littermates (*Tgfbr2*^+*/*+^) were collected and fixed at four different ages, in order to cover critical stages of postnatal development: (1) two weeks after birth (2w), which is before weaning and consumption of solid food; (2) six weeks after birth (6w), which is three weeks after the start of solid food consumption and still corresponds to a period of active growth; (3) twelve weeks after birth (12w), which corresponds to a late phase of skeletal growth; and (4) twenty-four weeks after birth (24w) when mice are skeletally mature. In human, these ages are analogous to 3-month-old, 11-year-old, 20-year-old and 30-year-old, respectively^21^. We collected a minimum of four males and four females from each genotype in each age group (Supplementary Table S1). Fixed skulls were then scanned using micro-CT and landmarked for geometric-morphometric analysis to compare the shape of the cranium and mandible between *Tgfbr2*^*G357W/*+^ and *Tgfbr2*^+*/*+^ mice (Supplementary Figures S1 and S2).

**Fig. 1:**
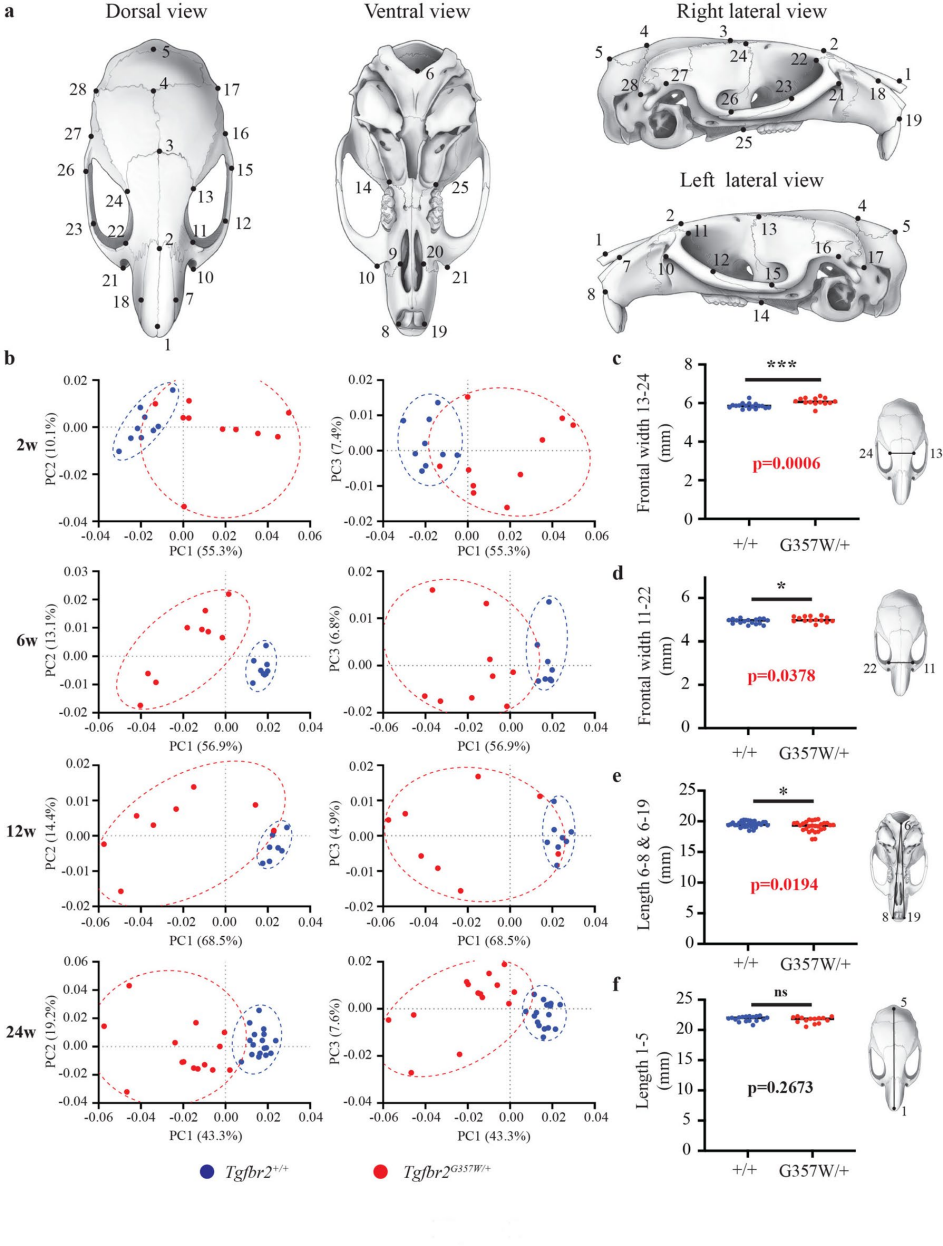
Landmarking of mouse cranium and analysis of shape variation in *Tgfbr2*^+*/*+^ and *Tgfbr2*^*G357W/*+^ mice. (**a**) Schematic representation of the mouse cranium from dorsal, ventral and lateral views, indicating the position of the 28 landmarks used for geometric morphometric analysis. The description of each landmark is shown in Supplementary Figure S1. (**b**) Principal component analysis (PCA) of cranial shape variation in *Tgfbr2*^+*/*+^ and *Tgfbr2*^*G357W/*+^ mice performed at 2 (2w), 6 (6w), 12 (12w) and 24 (24w) weeks after birth. Results are shown for the first three principal components (PC1, PC2 and PC3) and the percentage of variance contribution for each principal component is shown on the X and Y axes. (**c**) Quantification of the spacing between the two orbits measured between landmarks 13 and 24 at 24w. (**d**) Quantification of the spacing between the two orbits measured between landmarks 11 and 22 at 24w. (**e**) Quantification of the ventral length of the cranium measured between landmarks 6 and 8, and 6 and 19 at 24w. (**f**) Quantification of the dorsal length of the cranium measured between landmarks 1 and 5 at 24w.

Principal Component Analysis (PCA) of the cranium landmark 3D coordinates (Fig. 1a and Supplementary Figure S1) revealed a significant difference in cranial shape between *Tgfbr2*^*G357W/+*^ and *Tgfbr2*^+*/*+^ mice in all four age groups (Fig. 1b). Separation between the two genotypes based on skull shape was clearly visible. Visualization of the results of the PCA analyses, with Principal Component 1 (PC1) accounting for 55.3%, 56.9%, 68.5% and 43.3% of the variance at 2w, 6w, 12w and 24w, respectively, demonstrated a clear separation of the two groups, indicating remarkable differences between them. PC2 (10.1%, 13.1%, 14.4% and 19.2% of variance at 2w, 6w, 12w and 24w, respectively) and PC3 (7.4%, 6.8%, 4.9% and 7.6% of variance at 2w, 6w, 12w and 24w, respectively) axes did not significantly contribute to the separation of the group clusters. Moreover, while the *Tgfbr2*^+*/*+^ group formed a relatively tight cluster in the PCA plots, the *Tgfbr2*^*G357W/*+^ group exhibited a more spread-out distribution, indicating that the changes in cranial shape in *Tgfbr*^*G357W/*+^ mice are highly variable. The effect of genotype on cranial shape was significant across ages (*p* < 0.0001). In addition, the cranial centroid size of the *Tgfbr2*^*G357W/*+^ mice was significantly smaller than the *Tgfbr2*^+*/*+^ mice at all ages (*p* = 0.0022 at 2w, *p* = 0.0036 at 6w, *p* = 0.00186 at 12w, and *p* = 0.045 at 24w).

Despite the high morphological variability observed in *Tgfbr*^*G357W/*+^ mice, several distances between landmarks were significantly different between *Tgfbr2*^*G357W/*+^ and *Tgfbr2*^+*/*+^ mice at 24w: the width of the frontal bone between the two orbits, measured between the left and right frontal-squamosal intersections at the temporal crest (Fig. 1 c) and between the left and right intersections of frontal process of the maxilla with the frontal and lacrimal bones (Fig. 1d), was significantly larger in *Tgfbr*^*G357W/*+^ mice. This increase in orbital spacing was also found significant at 6w (Supplementary Figure S3). The ventral length of the skull, measured between the midpoints of the alveolar ridges above the upper incisors and point Basion, was significantly lower in *Tgfbr*^*G357W/*+^ mice at all four stages, even though it appeared more severe at earlier stages than at 24w (Fig. 1e and Supplementary Figure S3). Although the dorsal length of the skull between *Tgfbr2*^+*/*+^ and *Tgfbr*^*G357W/*+^ mice was not significantly different at 24w (Fig. 1f), it was significantly lower in *Tgfbr*^*G357W/*+^ mice at 2w, 6w and 12w (Supplementary Figure S3).

**Fig. 2:**
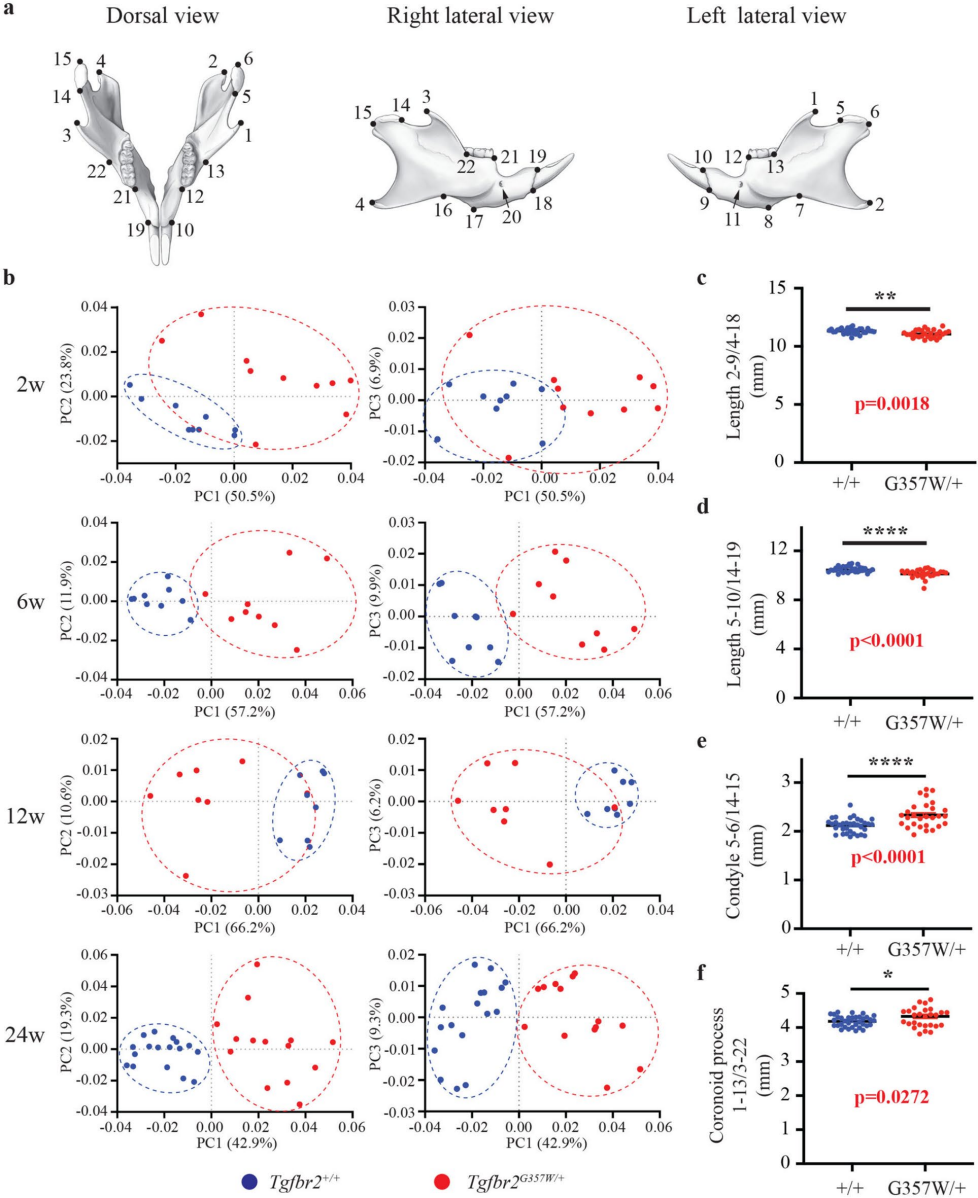
Landmarking of mouse mandibles and principal component analysis of shape variation in *Tgfbr2*^+*/*+^ and *Tgfbr2*^*G357W/*+^ mice. (**a**) Schematic representation of the mouse mandibles from dorsal and lateral views, indicating the position of the 22 landmarks used for geometric morphometric analysis. The description of each landmark is shown in Supplementary Figure S2. (**b**) Principal component analysis (PCA) of mandible shape variation in *Tgfbr2*^+*/*+^ and *Tgfbr2*^*G357W/*+^ mice performed at 2 (2w), 6 (6w), 12 (12w) and 24 (24w) weeks after birth. Results are shown for the first three principal components (PC1, PC2 and PC3) and the percentage of variance contribution for each principal component is shown on the X and Y axes. (**c**) Quantification of the mandible length measured between landmarks 2 and 9, and 4 and 18. (**d**) Quantification of the mandible length measured between landmarks 5 and 10, and 14 and 19. (**e**) Quantification of the condyle length measured between landmarks 5 and 6, and 14 and 15. (**f**) Quantification of the length of the coronoid process measured between landmarks 1 and 13, and 3 and 22.

The same analysis performed on the mandible (Fig. 2a and Supplementary Figure S2) provided similar results as the cranium analysis (Fig. 2b). PC1, which accounted for 50.5%, 57.2%, 66.2% and 42.9% of the variance at 2w, 6w, 12w and 24w, respectively, also indicated clear morphological differences between the two groups with the two group clusters clearly separating in the generated PCA plots. PC2 and PC3 axes did not significantly contribute to the separation, except at 2w when the two groups also exhibited separation along the PC2 axis. Similarly to what was demonstrated for the cranium, the *Tgfbr2*^*G357W/*+^ group exhibited a wider distribution when compared to the *Tgfbr2*^+*/*+^ group, indicating that the changes in mandibular shape in *Tgfbr*^*G357W/*+^ mice were also highly variable. The effect of genotype on mandibular shape was significant across ages (*p* < 0.0001). The mandibular centroid size of the *Tgfbr2*^*G357W/*+^ mice was smaller than the *Tgfbr2*^+*/*+^ mice at 2w (*p* = 0.0028), 6w (*p* = 0.0036), and 12 w (*p* = 0.0011), whereas the statistical difference was non-significant at 24w (*p* = 0.6668).

At 24w, the overall length of the mandible, measured between the incisor alveolar rim and both the mandibular angle (Fig. 2c) and the condyle bilaterally (Fig. 2d) was significantly reduced in *Tgfbr2*^*G357W/*+^ mice. This reduction was observed at all four developmental stages analyzed (Supplementary Figure S4). At 24w, the length of the condyle was significantly higher in *Tgfbr*^*G357W/*+^ mice (Fig. 2e). Interestingly, reduced condyle length was measured in *Tgfbr*^*G357W/*+^ mice at 2w, while increased condyle length was observed at 6w and 24w (Supplementary Figure S4). At 24 weeks, the length of the coronoid process was also significantly higher in *Tgfbr*^*G357W/*+^ mice (Fig. 2f). Interestingly, the coronoid process was significantly shorter in *Tgfbr*^*G357W/*+^ mice at 2w before catching up with *Tgfbr2*^+*/*+^ mice and becoming longer at 24w (Supplementary Figure S4).

These results indicate that variable changes in the shape of the cranium and mandible are associated with mutation in *Tgfbr2* in a mouse model for LDS2, which is consistent with our previous clinical observations ^8^. We further determined that these changes are present at a very young age, analogous to a 3-month-old baby. In terms of cephalometry, *Tgfbr*^*G357W/*+^ mice exhibited significant increase in the space between orbits, significant reduction in skull and mandible length, as well as significant increase in the length of the condyle and coronoid process.

### Volume registration confirms phenotypic variability and reveals craniofacial asymmetry in *Tgfbr2*^*G357W/*+^ mice

**Fig. 3:**
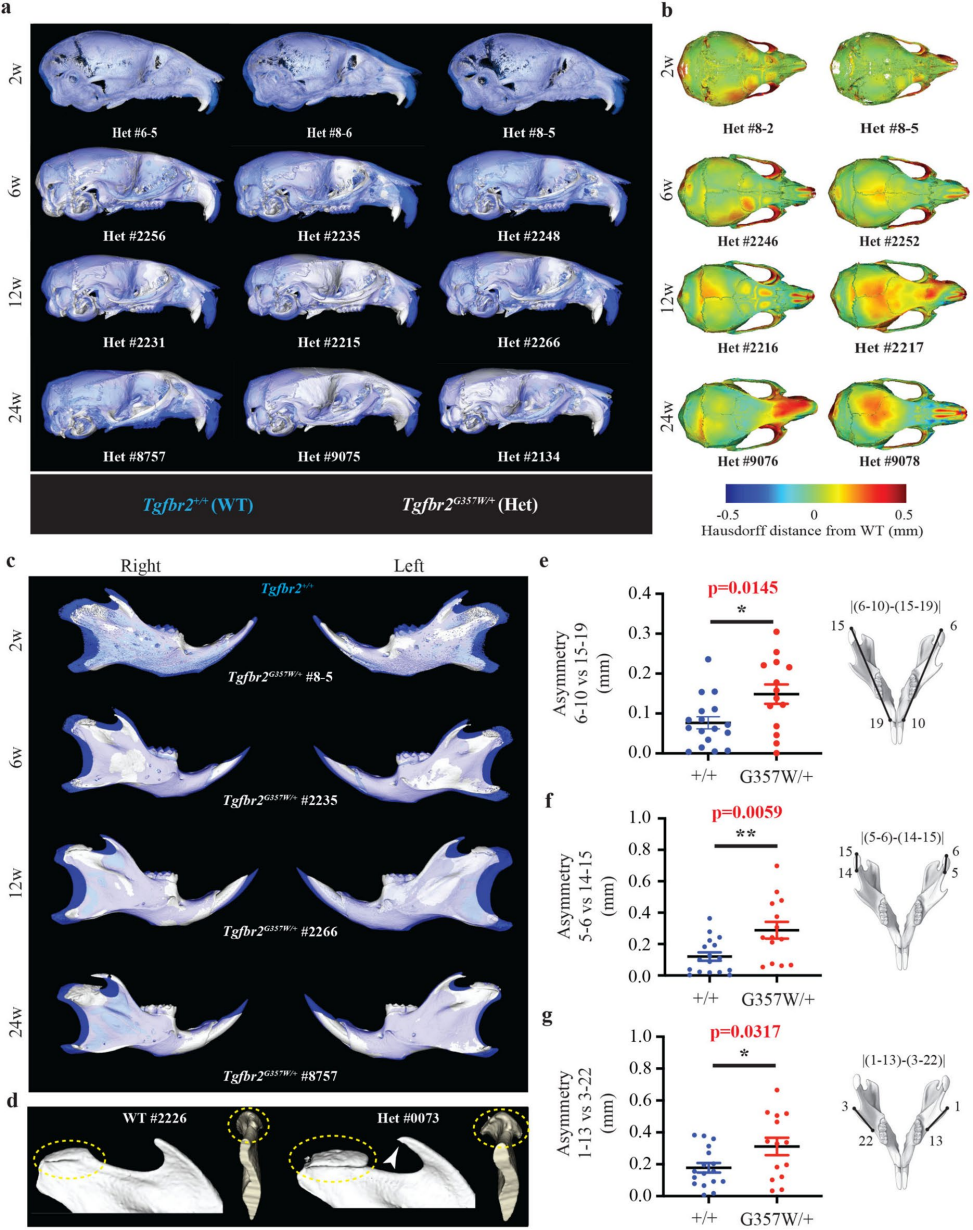
Registration of craniums and mandibles between *Tgfbr2*^+*/*+^ and *Tgfbr2*^*G357W/*+^ mice highlighting the asymmetry and high variability in shape variations. (**a**) Overlap of skulls from three *Tgfbr2*^*G357W/*+^ mice (white) to matched control skull (blue) at 2w, 6w, 12w and 24w. Rigid body surface registration was generated using the “CMF Reg” extension in 3D Slicer (https://www.slicer.org, open-source software). Note the various degrees of shortening of the anterior part of the skull while the posterior part of the skull tends to exhibit better alignment (**b**). Heatmaps highlighting the various degrees of deviation from the reference skull, shown for two skulls from *Tgfbr2*^*G357W/*+^ mice at 2w, 6w, 12w and 24w. Heatmaps were generated using the “Model to Model Distance” extension in 3D Slicer (https://www.slicer.org, open-source software). Note the various expressions of left–right asymmetry. (**c**) Overlap of mandibles from *Tgfbr2*^*G357W/*+^ mice to matched control mandibles at 2w, 6w, 12w and 24w. Note the shortening of the posterior part of the mandible (condyloid process, coronoid process and angle) while the anterior part of the mandible tends to exhibit better alignment, except for the incisor (**d**) High magnification view of the condyle and coronoid process of *Tgfbr2*^+*/*+^ and *Tgfbr2*^*G357W/*+^ mice showing severe condylar hyperplasia (yellow dashed ellipses) and protrusion of the coronoid process (white arrowhead) in mutant mice. (**e–g**) Quantification of the asymmetry between the left and right mandibles through measure of the absolute value of the difference in mandible length, between landmarks 6 and 10, and 15 and 19 at 24w (**e**), the absolute value of the difference in condyle length, between landmarks 5 and 6, and 14 and 15 at 24w (**f**), and the absolute value of the difference in the length of the coronoid process, between landmarks 1 and 13, and 3 and 22 at 24w (**g**).

To further characterize the type of morphological changes caused by mutation in *Tgfbr2* in the *Tgfbr2*^*G357W/*+^ mouse model, 3D volume registration was performed. Comparison of cranial shape between the two genotypes indicated that the frontal part of the cranium tends to be shorter in *Tgfbr2*^*G357W/*+^ mice when compared to *Tgfbr2*^+*/*+^ mice, and these morphological changes were observed as early as 2w, with variable degrees of anterior shortening (Fig. 3a). Heatmap representations of mutant craniums, highlighting the various degrees of deviation from control craniums, revealed variable expressions of left–right asymmetry in *Tgfbr2*^*G357W/*+^ mice (Fig. 3b). Comparison of mandibular shape revealed an overall shortening of the mandibles in *Tgfbr2*^*G357W/*+^ mice when compared to *Tgfbr2*^+*/*+^ mice, consistent with significant reduction in mandible length (Fig. 2, c and d), with the proximal part of the mandible (condyloid process, coronoid process and angle) exhibiting the strongest deviation (Fig. 3c). Deviation in the distal part of the mandible is mostly linked to differences in the shortening of the incisor^19^ while the mandibular symphysis showed better alignment. Two striking features observed in the mandible of *Tgfbr2*^*G357W/*+^ mice were severe hyperplasia of the condyle, consistent with increased condyle length described earlier, with osteophyte formation giving the condyle a mushroom-like shape deformity, and posterior extension of the coronoid process (Fig. 3d). Significant asymmetry between the right and left mandibles were observed when measuring the overall length between the incisor alveolar rim and the condyle (Fig. 3e), the length of the condyle (Fig. 3f), and the length of the coronoid process (Fig. 3g). Mandibular length asymmetry was also observed at 6w (Supplementary Figure S5). Significant left–right asymmetry was also observed in the maxilla at 6w and 12w (Supplementary Figure S5). Based on our 3D geometric morphometric analysis, we could confirm statistically significant differences in the degree of directional asymmetry between the two groups across all ages, for both the cranium and the mandible (*p* < 0.0001).

These data are consistent with the variability observed via 3D geometric morphometric analysis and with the cephalometric measurements. Moreover, our findings indicates that craniofacial asymmetry is frequently observed in *Tgfbr2*^*G357W/*+^ mice.

### Characteristic craniofacial anomalies were observed in *Tgfbr2*^*G357W/*+^ mice, some of which tend to be more prevalent in *Tgfbr2*^*G357W/*+^ females

**Fig. 4:**
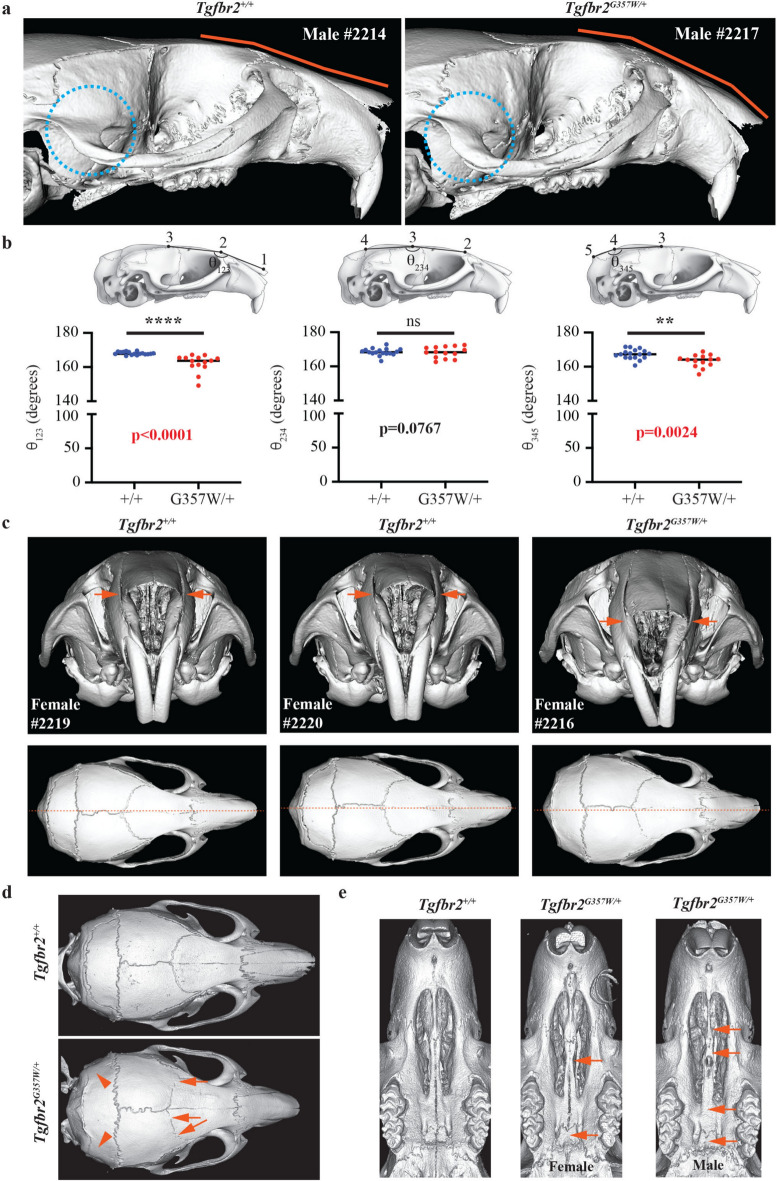
Characteristic craniofacial anomalies observed in *Tgfbr2*^*G357W/*+^ mice. (**a**) Lateral view of skulls from *Tgfbr2*^+*/*+^ and *Tgfbr2*^*G357W/*+^ mice showing severe cranial doming (orange lines) and sharp posterior curve of the zygomatic process of the temporal bone (blue dotted circles) in *Tgfbr2*^*G357W/*+^ mice at 12w. (**b**) Quantification of the angles between the nasal and frontal bones (θ_123_), the frontal and parietal bones (θ_234_), and the parietal and interparietal bones (θ_345_) at 12w and 24w (combined). Levels of statistical significance: **, p < 0.01; ****, p < 0.0001. p-values are indicated in red when significant. (**c**) Frontal view of skulls from two *Tgfbr2*^+*/*+^ mice and one *Tgfbr2*^*G357W/*+^ mouse showing mild (wild-type female #2220) and severe (mutant female #2216) asymmetry of the snout (orange arrows) at 12w. Lower panel images show a top view of the skulls highlighting deviation from the midline (orange dotted line), mild in wild-type female #2220 and severe in mutant female #2216. (**d**) Representative images showing cranial suture microfusions (orange arrows) and full fusions (orange arrowheads) at 24w. (**e**) Representative images showing palatal fusion (orange arrows) in *Tgfbr2*^*G357W/*+^ mice at 24w.

We identified a few characteristic anomalies that were commonly seen in *Tgfbr2*^*G357W/*+^ mice but absent in *Tgfbr2*^+*/*+^ mice. These included cranial doming (Fig. 4a), a phenotype that was corroborated by significant reduction in the angle between the frontal and nasal bones, while the angle between the frontal and parietal bones was unaffected (Fig. 4b). The angle between the parietal bones and the interparietal bone was also significantly reduced (Fig. 4b). These changes in the curvature of the skull are consistent with overall reduction in the ventral length of the skull (Fig. 1e) and shortening of the anterior part of the skull (Fig. 3a). Reduction in the angle between the nasal and frontal bone was observed at 6, 12 and 24w but not at 2w (Supplementary Figure S3).

**Fig. 5:**
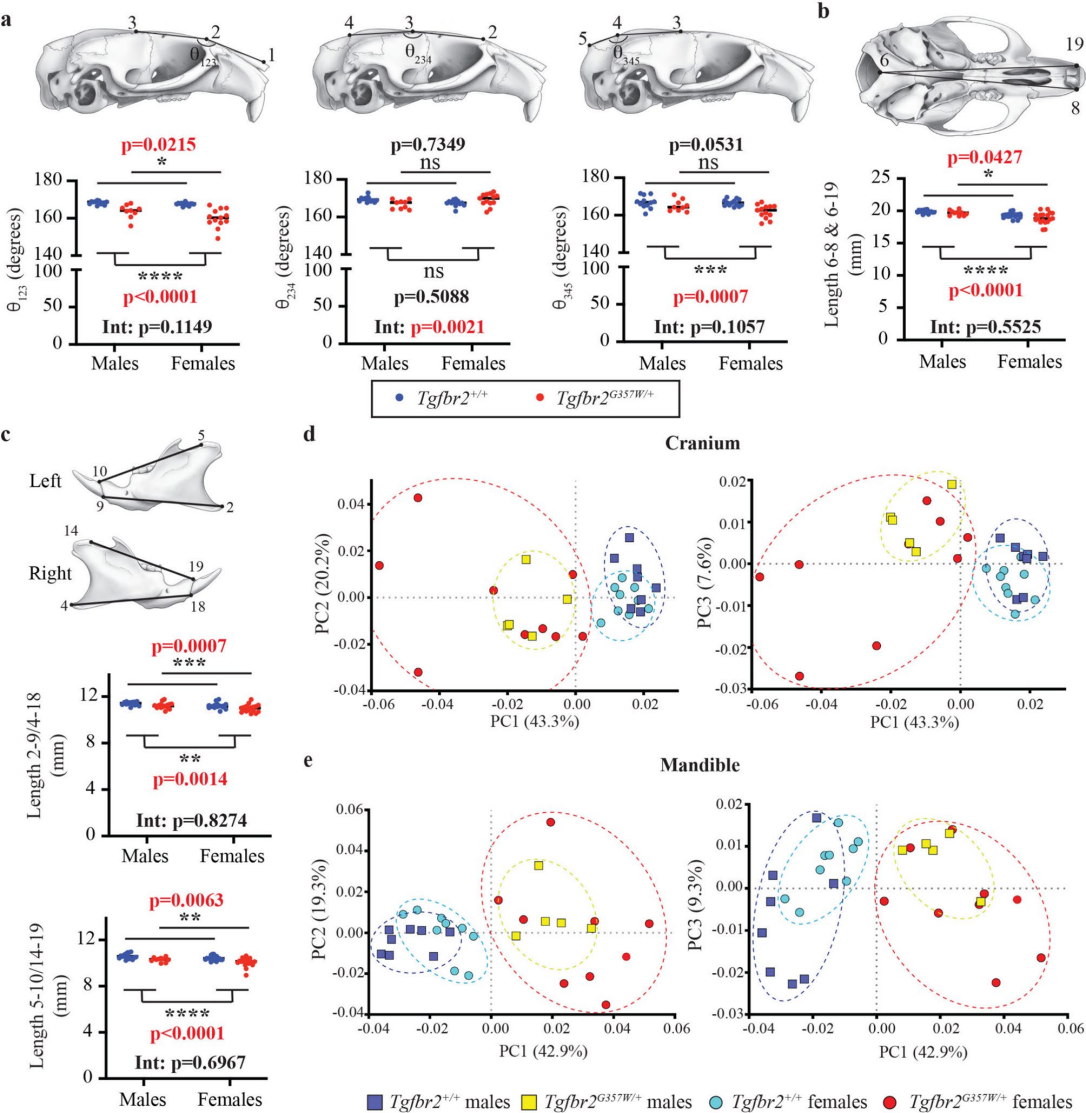
Difference in the severity of craniofacial anomalies between *Tgfbr2*^*G357W/*+^ males and females. (**a**) Two-way ANOVA comparison of the angles between the nasal and frontal bones (θ_123_), the frontal and parietal bones (θ_234_), and the parietal and interparietal bones (θ_345_), between male and female *Tgfbr2*^+*/*+^ and *Tgfbr2*^*G357W/*+^ mice. (**b**) Two-way ANOVA comparison of the ventral length of the skull measured between landmarks 6 and 8, and 6 and 19, between males and females *Tgfbr2*^+*/*+^ and *Tgfbr2*^*G357W/*+^ mice. (**c**) Two-way ANOVA comparison of the mandible length measured between landmarks 2 and 9, and 4 and 18, as well as between landmarks 5 and 10, and 14 and 19, between males and females *Tgfbr2*^+*/*+^ and *Tgfbr2*^*G357W/*+^ mice. Levels of statistical significance: *, p < 0.05; **, p < 0.01; ***, p < 0.001; ****, p < 0.0001. p-values are indicated on the graphs. Upper value correspond to sex effect while lower values correspond to genotype effect. The significance of interaction between sex and genotype in indicated at the bottom of each graph (Int). (**d**) PCA of cranial shape variation in *Tgfbr2*^+*/*+^ and *Tgfbr2*^*G357W/*+^ mice at 24 weeks (24w) after birth, as shown in Fig. 1b, with separation of males (squares) and females (circles). (**e**) PCA of mandible shape variation in *Tgfbr2*^+*/*+^ and *Tgfbr2*^*G357W/*+^ mice at 24 weeks (24w) after birth, as shown in Fig. 2b, with separation of males and females. Note that, for both cranium and mandibles, *Tgfbr2*^*G357W/*+^ females exhibit a more dispersed distribution and are more extremely separated from their wild-type counterparts when compared to males.

Other characteristic features included a more acute posterior curvature of the zygomatic process of the temporal bone (Fig. 4a), severe asymmetry of the nasal bones (Fig. 4c), and, as mentioned earlier, posterior extension of the coronoid process and condylar hyperplasia (Fig. 3d). Suture defects, in the form of microfusions or full suture fusion (Fig. 4d) in the cranium, as well as in the palate (Fig. 4e) were also observed. Interestingly, several of these features were not equally represented in males and females. Considering mice at 12w and 24w, cranial doming and severe nasal asymmetry of the snout appeared to be more frequently observed (visual assessment) in females, while the other features were equally represented in both sexes (Table 1 and Supplementary Table S2). Corroborating these sex differences, female *Tgfbr*^*G357W/*+^ mice exhibited a significantly more severe reduction in the angle between the nasal and frontal bone (Fig. 5a and supplementary Figure S6), as well as in the ventral length of the skull (Fig. 5b and supplementary Figure S6). Interestingly, mandibular length was also more significantly reduced in females *Tgfbr*^*G357W/*+^ than in males (Fig. 5c and Supplementary Figure S6). Overall, these sex differences were not present at 2w but developed at later stages (Supplementary Figure S6).

**Table 1:** Characteristic craniofacial anomalies observed at 12 + 24 weeks.

Craniofacial Anomalies	WT males (%, N = 12)	Het males (%, N = 9)	WT females (%, N = 13)	Het females (%, N = 13)	Fisher’s exact test (Het vs WT)
Cranial doming	0	44	0	77	p < 0.0001
Nasale asymmetry*	8	67	16	85	p < 0.0001
Upturned nasale	16	44	0	46	P = 0.0060
Sharp P-curve	0	66	7	69	p < 0.0001
Sharp coronoid process	0	60	0	44	p < 0.0001
Hyperplastic condyle	0	100	0	100	p < 0.0001
Localized suture fusions	8	22	0	38	p = 0.0180
Broad suture fusions	16	78	8	69	p < 0.0001
Palate fusion	16	100	0	100	p < 0.0001

Note: WT = *Tgfbr2*^+*/*+^ mice; Het = *Tgfbr2*^*G357W/*+^; P-curve = posterior curve of the zygomatic process of the temporal bone.

*Nasale asymmetry includes mild (commonly seen in wild-type mice) and severe (only seen in Hets). See Supplementary Table 2 for distinction mild/severe and for sex differences.

Based on the observed influence of sex on the presence of characteristic craniofacial anomalies in *Tgfbr2*^*G357W/*+^ mice, we revisited the PCA analysis by separating males and females. While both *Tgfbr2*^*G357W/*+^ males and *Tgfbr2*^*G357W/*+^ females clearly separated from their WT counterparts, *Tgfbr2*^*G357W/*+^ females separated more substantially from *Tgfbr2*^+*/*+^ females and exhibited a more expanded distribution when compared to *Tgfbr2*^*G357W/*+^ males for both the cranium (Fig. 5d) and the mandibles (Fig. 5e). Given the small sample size when separating sexes, GMA statistical analysis lacked power. These results indicated no substantial sexual dimorphism in the cranial shape of *Tgfbr2*^*G357W/*+^ mice across the age stages (*p* = 0.9274 at 2w, *p* = 0.563 at 6w, *p* = 0.5774 at 12w and *p* = 0.6176 at 24w). No significant sexual dimorphism was detected in the shape of the mandible either (*p* = 0.4206 at 2w, *p* = 0.563 at 6w, *p* = 0.5514 at 12w, *p* = 0.2348 at 24w). The effect of sex on the centroid size was not significant across age stages either for the cranium (*p* = 0.8807 at 2w, *p* = 0.5547 at 6w, *p* = 0.2447 at 12w, and *p* = 0.0965 at 24w) and mandible (*p* = 0.83 at 2w, *p* = 0.6606 at 6w, *p* = 0.0624 at 12w, and *p* = 0.5626 at 24w). However, the effect of sex on the degree of asymmetry for the cranium was statistically significant in the 12w (*p* = 0.0157) group and 24w (*p* < 0.0001) group, whereas it appeared as non-significant in the 2w (*p* = 0.9503) and 6w (*p* = 0.5572) groups. The effect of sex on mandibular asymmetry was significant in the 2w (*p* = 0.0015), 6w (*p* < 0.0001), and 24w (*p* = 0.0028) groups, whereas it was non-significant in the 12w group (*p* = 0.0874).

Taken together, these findings indicate that mouse craniofacial development is not equally affected by mutation in *Tgfbr2* in males and females, with females tending to exhibit higher severity, variability and asymmetry.

### Female patients with LDS2 also tend to exhibit more severe and variable craniofacial anomalies than males

**Fig. 6:**
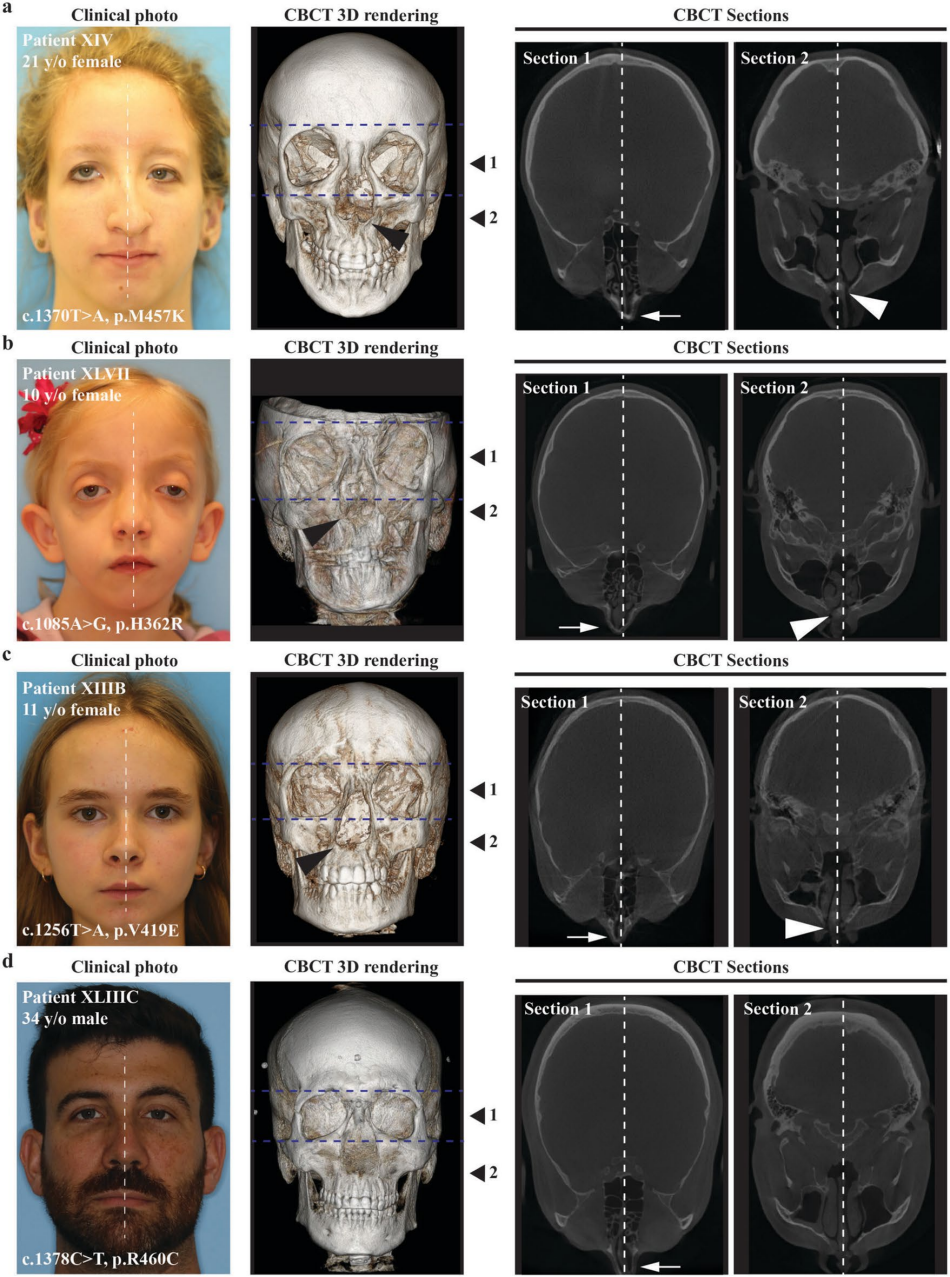
Severe deviation of the nasal tip and abnormal eye shape in females with LDS2. (**a**) Clinical photo, CBCT 3D reconstruction and section images from a 21-year-old female with *TGFRB2*:c.1370 T > A mutation resulting in a methionine-to-lysine substitution at amino acid 457 (p:M457K) in the protein kinase domain of the receptor. Note the severe deviation of the nasal tip (white arrow) and the skeletal deviation of the nasal bone and the base of the nasal cavity (black and white arrowheads). Abnormal eye shape is due to misalignment of the orbits (blue dotted lines). The positions of Sect. 1 and Sect. 2 are indicated on the right side of the CBCT 3D rendering. (**b**) Same as A for a 10-year-old female with *TGFBR2*:c.1085A > G mutation resulting in a histidine-to-arginine substitution at amino acid 362 (p.H362R) in the protein kinase domain of the receptor. (**c**) Same as A for an 11-year-old female with *TGFBR2*:c.1256 T > A mutation resulting in a valine-to-glutamate substitution at amino acid 419 (p.V419E) in the protein kinase domain of the receptor. Contrary to A and B, this patient exhibits milder deviation of the nasal tip and no eye shape abnormalities. (**d**) Same as A for a 34-year-old male with *TGFBR2*:c.1378C > T mutation resulting in an arginine-to-cysteine substitution at amino acid 460 (p.R460C) in the protein kinase domain of the receptor. This male patient exhibits normal eye shape and mild deviation of the nasal tip when compared to A and B. Skeletal assessment indicates mild deviation of the nasal bone with no deviation at the base of the nasal cavity.

In our human cohort, sixteen female and ten male patients of various ages (2.6–57.4 years) with LDS2 were included (Supplementary Table S3). Considering the sexual dimorphism observed in mice, we revisited our updated human cohort data to determine if there was any indication that females may be more severely affected than males and exhibit unique features. Based on clinical evaluation, fourteen patients had mild to moderate vertical orbital dystopia (14/26; 53.8%), which was equally prevalent between males and females (Supplementary Table S4; Fig. 6, a and b). Downslanting palpebral fissures were observed in eighteen patients (18/26; 69.2%), most of which were females (61%). In addition, eight patients presented with deviation of the nose (30.7%), five of which were females (62.5%).

**Fig. 7:**
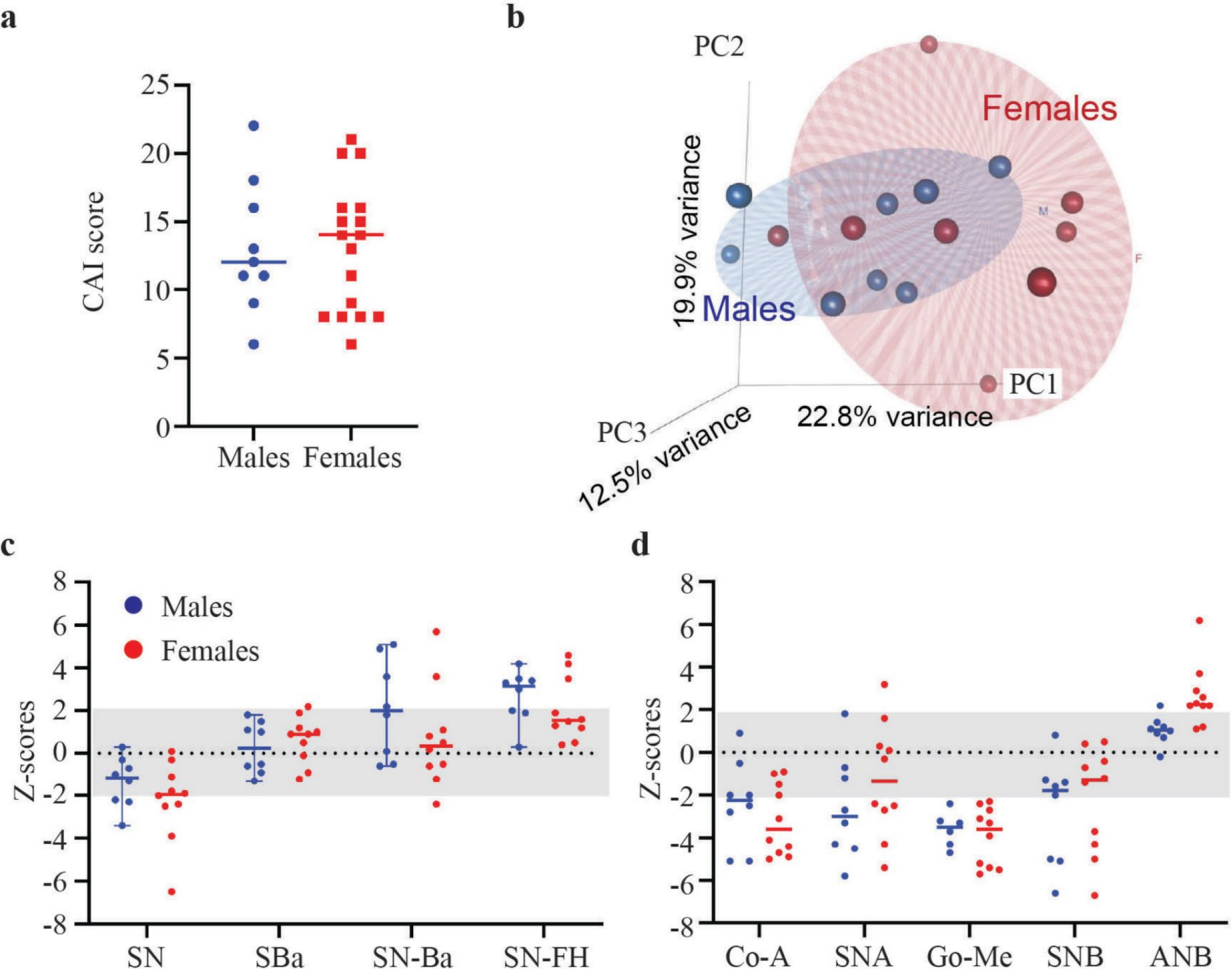
Severity and variability of the craniofacial phenotype in patients with LDS2. (**a**) Scatter plot graph summarizing the Craniofacial Abnormality Index (CAI) scores of the LDS2 human cohort. The bar marks the median value. Note that females tend to have a greater score than males, indicating a higher prevalence of craniofacial differences that are more prevalent in LDS subjects. (**b**) Screenshot of a 3D PCA plot depicting the overall craniofacial shape variation in patients with LDS2. Note that female patients present with a more variable craniofacial shape in comparison to males, including more outliers. (**c**) Scatter plots summarizing the results of the cephalometric analyses for measurements related to the cranial base. SN, Sella-Nasion distance; SBa, Sella-Basion distance; SN-Ba, angle between the Sella-Nasion (SN) and the Basion-Nasion lines; SN-FH, angle between the Sella-Nasion line and the Frankfort Horizontal plane. A shorter mean anterior cranial base length, as well as increased cranial base angle and SN-FH measurements in comparison to the general population normative values can be appreciated. (**d**) Scatter plots summarizing the results of the cephalometric analyses for measurements related to the maxilla and mandible. The grey bar area depicts the standard range of statistical clinical significance (Z-score ± 2 SD from the norm). Co-A, Condylion-A-point distance; SNA, Sella-Nasion-A-point angle; GoMe, Gonion-Menton distance; SNB, Sella-Nasion-B-point; ANB, relative position of the maxilla to the mandible (SNA-SNB). A shorter midface length and reduced maxillary projection, shorter mandible and reduced mandibular projection, as well as increased skeletal intermaxillary sagittal discrepancy can be appreciated. The skeletal discrepancy was more severe in females (*p* = 0.01).

2D photos and 3D CBCT reconstructions data were evaluated for three females and one male with nasal deviation (Fig. 6). For the three females considered, the CBCT data indicated that deviation of the nasal tip was due to underlying skeletal asymmetry involving the nasal bone as well as the base of the nasal cavity (Fig. 6, a-c) and not just due to deviation of the nasal septum (cartilage and soft issue). This finding is consistent with the severe nasal bone asymmetry observed in the *Tgfbr*^*G357W/*+^ mice. The male exhibited a milder clinical phenotype when compared to the three females, with no deviation at the base of the nasal cavity (Fig. 6d). Based on the clinical evaluation it was also appreciated that sixteen patients presented with midface flatness (61.5%), most of which were females (68.8%). Respectively, mandibular hypoplasia was noted for twenty-two patients (84.6%), fifteen of which were females (68.2%). Upon calculation of the Craniofacial Abnormality Index (CAI) which has been previously developed for LDS patients by our group^8^, the score tended to be higher for the females (CAI = 14) than for the males (CAI = 12) (Fig. 7a), indicating a trend for greater prevalence of craniofacial anomalies in females versus males.

The PCA explained 55.3% of the craniofacial shape variation in the first three PCs (Fig. 7b). There was statistically significant directional asymmetry of the overall craniofacial shape (*p* < 0.0001), as well as a significant effect of sex on the craniofacial shape (*p* < 0.0001). Based on the generated PCA plot, there is increased variability of the overall craniofacial shape in females in comparison to males, with more female extreme phenotypes, who appear as outliers mainly across the PC2 axis. There was no statistically significant difference of the centroid size between male and female patients (*p* = 0.5240).

The results of the cephalometric analyses confirmed the results of our previous study. Specifically, pertinent positive results included the presence of a shorter anterior cranial base length (SN), which was more prevalent in females. Increased cranial base angle (SN-Ba) was more prevalent in males, with two female outliers. An increased SN-FH angle was also noted in several patients, which has been attributed to a lower-than-average anatomical position of landmark Sella (Fig. 7c). Decreased midface length (Co-A) and maxillary projection (SNA) were observed overall, which agrees with the high prevalence of midface flatness that was appreciated during the clinical evaluation. Although the craniofacial anatomy of humans and mice differs considerably, this finding could be considered consistent with the cranial doming in the *Tgfbr*^*G357W/*+^ mice, which results in a decreased projection of the anterior part of their skull. The length of the mandible (GoMe) and the mandibular projection (SNB) were also decreased on average, which is consistent with the reduced mandible length in the *Tgfbr2*^*G357W/*+^ mice. Finally, the skeletal discrepancy between the maxilla and mandible was increased overall, mostly in females (ANB) (Fig. 7d).

## Discussion

One of the major limitations faced when studying craniofacial shape variation in human disease cohorts is the lack of skeletal 3D-imaging data from very young patients. Due to the risks related to the exposure of children to radiation^22^, primarily related to the stochastic risk of radiation-induced carcinogenesis, the acquisition of skeletal 3D images in the form of CT or CBCT scans is usually only justified in cases where there is a clinical indication to do so. We encountered the same limitation when studying craniofacial shape variations in a cohort of patients with LDS (Almpani et al., 2021). Skeletal craniofacial imaging in infants is also clinically indicated even more rarely for the same reason. It was, therefore, difficult to evaluate if the craniofacial shape variations associated with LDS were present at birth or if they developed during postnatal growth. In this study, with the use of a mouse model for LDS2, 3D geometric-morphometric analysis revealed that craniofacial shape in LDS2 significantly deviates from normal craniofacial shape as early as two weeks after birth, which corresponds to three months in humans. This result strongly indicates that these variations are present at birth and do not occur during postnatal mouse development, which may also be the case in patients.

Another inherent limitation in the case of rare diseases is the small cohort size, usually combined with broad age range and genetic heterogeneity. In our previous human cohort study, we observed a high variability in the penetrance and expressivity of the craniofacial anomalies associated with LDS, which could have been attributed to the impact of genetic modifiers and/or environmental factors. In the present study using *Tgfbr2*^+*/*+^ mice, despite the pure genetic background of the mouse model used, a wide variability in the way craniofacial shape is affected was also observed. Significant craniofacial asymmetry was also observed in the mouse model. This indicates that the phenotypic variability observed in LDS2 is, at least in part, inherently related to the mode of action of the mutation on tissue development, independent of genetic modifiers that may also have an additive effect. The phenotypic variability observed in this model contrasts with the uniform and consistent craniofacial phenotype (100% full cleft palate, 100% shortening of the mandible) observed in all the conditional knockout models previously used to study the function of *Tgfbr2* in craniofacial development^12–15^. Most importantly, it recapitulates the phenotypic variability observed in humans affected by LDS2.

Apart from overall shape variations, we also identified specific craniofacial features that are consistent with traits observed in patients with LDS, including variable suture defects observed both in the cranium (consistent with variable craniosynostosis observed in patients) and in the palate (consistent with variable high arched palate observed in patients), abnormal condylar shape (consistent with temporomandibular joint abnormalities observed in patients), and variable reduction in mandibular size (consistent with variable degree of mandibular hypoplasia observed in patients). Interestingly, several of these features were more prevalent in females than in males, and overall shape variation also showed a more dispersed distribution in females than in males, suggesting sexual dimorphism in craniofacial anomalies in LDS. Although cohort size limits our ability to corroborate this sex difference in patients with LDS, our revisited human data tend to support this hypothesis. Interestingly, a recent study also revealed differences between males and females in the expression of the cardiovascular phenotype in LDS^23^.

The animal model for LDS2 used in this study recapitulates various aspects of the craniofacial phenotype observed in patients with LDS2, including the variability of the phenotype and potential sex differences. Future work will determine if the severity of craniofacial anomalies could be used as a prognostic tool for cardiovascular risk, which is also highly variable in LDS. Moreover, the animal model will be used to elucidate the mechanism of action of the mutation in *Tgfbr2* on the development of various elements of the skull. Finally, it will provide new molecular insights that will be essential for the development of potential therapeutic approaches for the treatment of craniofacial anomalies in LDS.

## Materials and methods

### Animal model

A knock-in murine model heterozygous for the p.G357W mutation in the *Tgfbr2* gene that causes LDS2 was obtained^17^. *Tgfbr2*^*G357W/*+^ mice were bred with wild type 129S6 mice (inbred line) to generate the adequate number of *Tgfbr2*^*G357W/*+^ mice and wild-type littermates (*Tgfbr2*^+*/*+^ mice) needed for the study. Four postnatal age groups of *Tgfbr2*^*G357W/*+^ and *Tgfbr2*^+*/*+^ mice were collected: 2 weeks, 6 weeks, 12 weeks, and 24 weeks. Breeding and collection continued until each age group amassed a minimum of 4 males and 4 females (Supplementary Table S1). All procedures on animals were approved by the NIDCR Animal Care and Use Committee and follow the ARRIVE Guidelines. All methods were performed in accordance with the relevant guidelines and regulations.

### Specimen preparation

Mice were euthanized using a CO_2_ chamber followed by cervical dislocation. To prepare each subject for analysis, heads were dissected and the skin surrounding the skull was excised. The skulls were then fixed in a 4% solution of Paraformaldehyde (PFA) powder diluted with 1X Phosphate-Buffered Saline (PBS), pH 7.4. From the time of initial submersion in 4% PFA fixative, the skulls were washed with 1X PBS at 48 h, then washed again at 72 h, and finally stored in 70% ethanol at 96 h.

### 3D imaging

Full-skull 3D micro computed tomography (microCT) images were then taken of each mouse using the μCT 50 specimen scanner (Scanco Medical AG, Bassersdorf, Switzerland) at 70 kV X-ray source voltage, 85 μA of intensity/beam current, power at 6 W, with 0.5 mm Al filter, collecting 2,000 projections in a 360-degree rotation (0.18-degree rotation) at 900 ms integration time. The image resolution was 10 μm for skulls at 2 weeks and 17 µm for skulls at 6 weeks, 12 weeks, and 24 weeks. Quality checks were performed for each microCT scan to ensure the entire skull was imaged and clearly visible for analysis. These images were then converted into DICOM files using the program AnalyzePro 1.0 (AnalyzeDirect, Inc., Overland Park, KS, United States).

### 3D analysis

#### Landmark annotation

The DICOM files were imported into Anatomage InVivo6.5 imaging software, where 50 landmarks were placed on each subject by one trained and calibrated user. 49 of these landmarks were previously defined for murine landmark analysis^24^. One additional landmark, Basion, was placed at the median (midline) point of the anterior margin of the foramen magnum of the cranium, as a representative point of the most posterior part of the cranial base. 28 landmarks were annotated on the cranial (nasal, frontal, parietal, interparietal, occipital, temporal, sphenoid), zygomatic, and maxillary craniofacial bones. 22 landmarks were annotated on the mandible. The Euclidean coordinates generated were exported as.csv files.

#### 3D volume registration and heatmap generation

Micro-CT images were segmented to separate the cranium from the mandible with the use of the object separator tool in AnalyzePro. Qualitative morphology comparisons for the cranium and mandible separately were made using extensions in 3D Slicer (https://www.slicer.org, open-source software). Rigid body surface registration was used via the extension “CMF Reg” to align samples and heatmap visualization was performed using the “Model to Model Distance” extension.

#### Quantitative measurement based on 3D landmark coordinates

To calculate the distance (*d*) between two points *A* = (X1,Y1,Z1) and *B* = (X2,Y2,Z2) in a 3D volume, the following formula was used:

$$d = \sqrt {\left( {X2 - X1} \right)^{2} + \left( {Y2 - Y1} \right)^{2} + \left( {Z2 - Z1} \right)^{2} }$$

To calculate the angle between three points *A* = (X1,Y1,Z1), *B* = (X2,Y2,Z2) and *C* = (X3,Y3,Z3) in a 3D volume (angle of vertex B), the following steps were followed:

(1) Calculate vectors $\overrightarrow{BA}$ and $\overrightarrow{BC}$:

$$\overrightarrow {BA} = A - B = \left( {X1 - X2, Y1 - Y2,Z1 - Z2} \right)$$

$$\overrightarrow {BC} = C - B = \left( {X3 - X2, Y3 - Y2,Z3 - Z2} \right)$$

(2) Calculate the dot product of $\overrightarrow{BA}$ and $\overrightarrow{BC}$

$$\overrightarrow {BA} \cdot \overrightarrow {BC} = \left( {X1 - X2} \right)\left( {X3 - X2} \right) + \left( {Y1 - Y2} \right)\left( {Y3 - Y2} \right) + \left( {Z1 - Z2} \right)\left( {Z3 - Z2} \right)$$

(3) Calculate the magnitude of $\overrightarrow{BA}$ and $\overrightarrow{BC}$

$$\parallel \overrightarrow {BA} \parallel = \sqrt {\left( {X1 - X2} \right)^{2} + \left( {Y1 - Y2} \right)^{2} + \left( {Z1 - Z2} \right)^{2} }$$

$$\parallel \overrightarrow {BC} \parallel = \sqrt {\left( {X3 - X2} \right)^{2} + \left( {Y3 - Y2} \right)^{2} + \left( {Z3 - Z2} \right)^{2} }$$

(4) Calculate the cosine of the angle *q* between $\overrightarrow{BA}$ and $\overrightarrow{BC}$

$$\cos \theta = \frac{{\overrightarrow {BA } \cdot \overrightarrow {BC} }}{{\parallel \overrightarrow {BA} \parallel \cdot \parallel \overrightarrow {BC} \parallel }}$$

(5) Calculate the angle *q* (in radian)

$$\theta \left( {radians} \right) = \arccos \left( {\cos \left( \theta \right)} \right)$$

(6) Convert the angle to degrees

$$\theta \left( {\deg rees} \right) = \theta \left( {radians} \right) \cdot \left( {\frac{180}{\pi }} \right)$$

#### Macroscopic phenotyping

Notable skeletal craniofacial features were recorded. The frequency of these characteristics was determined to compare the prevalence of these anomalies between the *Tgfbr2*^*G357W/*+^ mice and their wild type littermates.

#### Human cohort data

The craniofacial evaluation of a cohort of twenty-six patients (61.5% female) with a genetic diagnosis of LDS2 was also included in this study to validate the animal model-based analyses. The craniofacial deep phenotyping of fourteen patients included in the present cohort have been previously published^8^. The demographic characteristics of the updated cohort are included in Supplementary Table S3. The patients were enrolled at the National Institutes of Health (NIH) between 2015 and 2025 and have been consented onto Institutional Review Board (IRB) approved protocols (NCT02639312, Principal Investigator: Lee; NCT02504853, Principal Investigator: Guerrerio). All experimental protocols were approved by the IRB, and all methods were carried out in accordance with relevant guidelines and regulations listed in the approved protocols. A signed informed consent was obtained from all subjects and/or their legal guardian for the participation in the study and the publication of clinical images.

As part of this study, all patients underwent a thorough craniofacial evaluation which included thorough clinical phenotyping, facial, and intraoral photographs. All procedures took place at the dental clinic of the NIH Clinical Center. The Craniofacial Anomalies Index (CAI), previously developed by our group for the craniofacial evaluation of patients with LDS^8^, was also calculated to facilitate the comparison between males and females.

A total of 18 full head CBCT scans were also acquired (Planmeca ProMax 3D system, Planmeca, Helsinki, Finland; low dose mode ~ 30 μSv; 400 µm resolution)^8^. The characteristics of the patients with CBCT scans are also included in Supplementary Table S3. A validated set of 3D landmarks were annotated on the 3D CBCT volumes^25^ with the use of Invivo7 software (Anatomage, Santa Clara, CA, USA). Cephalometric analyses were conducted based on linear and angular measurements between anatomical landmarks that have been previously validated in the literature and compared normative values for the general population measured using the same software. Specifically, the length of the anterior (Sella-Nasion distance; SN) and posterior cranial base (Sella-Basion distance—SBa) and the cranial base angle (Nasion-Sella-Basion; SN- Ba), the projection of the maxilla (Sella- Nasion- Apoint angle—SNA) and the mandible (Sella- Nasion- Bpoint angle; SNB), and their relative projections in the sagittal plane (A point- Nasion- B-point; ANB), as well as the lengths of the maxilla (Condylion- Apoint distance; CoA) and mandibular body(Gonion- Menton distance; GoMe) were measured. The angle between the anterior cranial base and the Frankfort Horizontal plane was also included (Sella-Nasion plane/Porion-Orbitale plane; SN/FH). The z-scores (number of standard deviations from the normative values) were calculated for each measurement.

### Statistical analyses

The.csv files containing the 3D coordinate data were imported into the program MorphoJ for geometric morphometric analysis (GMA)^26^. A Procrustes Superimposition optimally aligned the imported coordinates through scaling, translation and rotation. The centroid size (CS) of each animal’s cranium and mandible was calculated as the square root of the sum of the squared inter-landmark distances measured from all mandibular landmarks to the centroid of the configuration and was used as an indicator of size. The CS data were tested for normal distribution and equality of variance by Shapiro–Wilk’s test and Levene’s test, respectively. The difference between the mean CS values of at all *Tgfbr2*^*G357W/*+^ versus *Tgfbr2*^+*/*+^ craniums and mandibles at all four age stages was tested by independent t-test or Mann–Whitney U for parametric and non-parametric data distributions respectively.

Principal Component Analyses (PCAs) were conducted for the exploration of the craniofacial morphological variation of *Tgfbr2*^*G357W/*+^ versus *Tgfbr2*^+*/*+^ craniums and mandibles in each age group. Procrustes analysis of variance (Procrustes ANOVA) Procrustes was used for the assessment of directional asymmetry, as well as for the effects of sex in shape and centroid size in the dataset. GraphPad Prism software was used for the generation of PCA graphs that were used for visualization and interpretation of the results.

For the human cohort the Euclidian coordinate values of the landmarks annotated on the CBCT scans were also used to conduct GMA. Two CBCT scans of female patients had to be excluded from this analysis due to significant motion artifacts. The same methodology described above for the mouse skull-based coordinate values was followed with the addition of an age-based regression analysis to account for the effect of ontogeny on the cohort. RStudio (v. 2024.09.0, Posit PBC, Boston, MA, USA) platform was used for the 3D visualization of the results of the PCA analysis^27^. Procrustes analysis of variance (Procrustes ANOVA) analysis was performed for the assessment of directional asymmetry in the dataset. The sample size in both cohorts was small relative to the dimensionality of the data. Therefore, GMA derived results are subjected to sampling error.

All other statistical analyses (unpaired t-tests, two-way ANOVAs, Fisher’s exact tests, and Chi-square analyses) were performed using Prism (GraphPad Software, Inc., la Jolla, CA, USA). All data are presented as Mean ± SEM. Statistical significance is indicated on graphs by *: *, *p* < 0.05; **, *p* < 0.01; ***, *p* < 0.001; ****, *p* < 0.0001. *p*-values are also indicated on graphs.

## Supplementary Information

Supplementary Information.

## Data Availability

All data needed to support the findings of this study are available within the manuscript and its supplementary information files.
