# Supplementary material for: Variability, asymmetry and sexual dimorphism in craniofacial anomalies in Loeys-Dietz syndrome 2: geometric morphometric analysis in mice
Source: Sci Rep. 2026 Jan 10;16:2185. doi: 10.1038/s41598-026-35325-8 (PMC12811269; doi:10.1038/s41598-026-35325-8)
Supplement: Supplementary file 1 — Supplementary Information. [file 41598_2026_35325_MOESM1_ESM.pdf]

Supplementary Table S1: Number of mice used for geometric morphometric analysis

Age	Female WT	Female Het	Male WT	Male Het	Total
**2 weeks**	5	6	4	4	19
**6 weeks**	5	5	4	4	18
**12 weeks**	4	4	4	4	16
**24 weeks**	9	9	8	5	31
**Total**	23	24	20	17	84

Note: WT = *Tgfbr2*$^{+/+}$ mice; Het = *Tgfbr2*$^{G357W/+}$ mice

Supplementary Table S2: Nasal asymmetry observed at **12+24 weeks**

**Nasale Asymmetry**	**WT males (%, N=12)**	**Het males (%, N=9)**	**WT females (%, N=13)**	**Het females (%, N=13)**	**Chi-square test WT (female vs male)**	**Chi-square test Het (female vs male)**
**Unaffected**	92	33	85	15		
**Mild**	8	56	15	39	P=0.5578	P=0.0827
**Severe**	0	11	0	46	(mild vs severe)	(severe vs other)

Note: WT = *Tgfbr2*$^{+/+}$ mice; Het = *Tgfbr2*$^{G357W/+}$

Supplementary Table S3. Summary of the demographic characteristics of the human subject LDS2 cohort.

**Clinical Assessment**		**CBCT Assessment**	
Characteristic	***Patient No***	***Characteristic***	***Patient No***
**Sex**		**Sex**	
Males	10	Males	8
Females	16	Females	10
Total	26	*Total*	18
**Age**		**Age**	
Range	2.6-57.4	Range	7-57.4
Average	20.4	Average	26.2
**Race***		**Race***	
White- European descent	18	White- European descent	13
Multiple races	3	Multiple races	2
Black- African American	2	Black- African American	1
White -Hispanic	2	White -Hispanic	1
Asian - East and South	1	Asian - East and South	1

**Self-reported*

Supplementary Table S4. Prevalence of craniofacial anomalies in male and female in patients with LDS2

Patient ID	Genetic Variant	Age	Sex	Vertical eye dystopia	Down-slanted palpebral fissures	Nose asymmetry	Midface flatness	Mandibular retrognathism
**IV**	c.1279>G, p.P426A	12.9	M	+	+	+	+	-
**XIIIA***	c.1256T>A, p.V419E	44.3	M	-	+	-	+	+
**XIIIB***	c.1256T>A, p.V419E	11.1	F	-	-	+	+	+
**XIV**	c.1370T>A, M457K	21.6	F	+	+	+	+	+
**XV**	c.1277C>A, p.A426D	7.1	F	-	+	+	+	+
**XVI**	c.1376T>A; p.M459K	10.7	M	+	-	-	-	+
**XVII****	c.1583G >A, p.R528H	17.3	F	+	-	-	+	-
**XVIII****	c.1583G >A, p.R528H	11.2	M	+	+	-	+	+
**XIX****	c.1583G >A, p.R528H	13.3	F	-	+	+	+	+
**XX**	c.985G>A, p.A329T	16.1	F	-	+	-	+	+
**XXI****	c.1583G>A, p.R528H	3.2	F	-	+	-	+	+
**XXII**	c.1190A>G, p.D397G	8.9	M	+	+	-	+	+
**XXIII**	c.1336G>A, p.D446N	12.6	F	+	+	-	+	+
**XXIV**	c.1483C>G; p.R495G	57.4	F	+	+	-	+	+
**XXV**	c.1583G>A, p.R528H	12.4	F	-	+	-	-	+
**XLIIIA*****	c.1378C>T, p.R460C	39.4	F	-	-	-	-	+
**XLIIIB*****	c.1378C>T, p.R460C	37.7	F	-	+	-	-	+
**XLIIIC*****	c.1378C>T, p.R460C	34.5	M	-	+	+	-	+
**XLIV**	c.902A>G, p.H301R	7	M	+	+	-	+	+
**XLV**	c.791G>A, p.R254H	32.3	F	+	+	-	-	+
**XLVI**	c.2819G>T, p.G940V	54.8	F	+	-	-	-	+
**XLVII**	c.1085A>G, p.H362R	10.8	F	+	+	+	+	+
**XLVIII**	c.1598G>A, p.C533Y	42.9	M	+	-	+	-	-
**XLIX**	c.1010T>G, p.L337R	3.1	M	+	-	-	-	+
**L**	c.1582 C>T, p.R528C	4.3	F	-	+	-	+	+
**LXIII**	c.1570G>A, p.D524N	2.6	M	-	+	-	-	-
**Total Cohort %**				**53.8%**	**69.2%**	**30.7%**	**61.5%**	**84.6%**
Male%				50%	49%	37.5%	31.2%	31.8%
Female%				50%	**61%**	**62.5%**	**68.8%**	**68.2%**

Notes:
Presence is noted with a “+” symbol and absence is noted with a “-“ symbol.
*Patient XIIIA is the father of patient XIIIB.
**Patients XVII, XVIII, XIX and XXI have the same genetic variant but are unrelated.
***Patients XLIIIA, XLIIIB and XLIIIC are siblings.

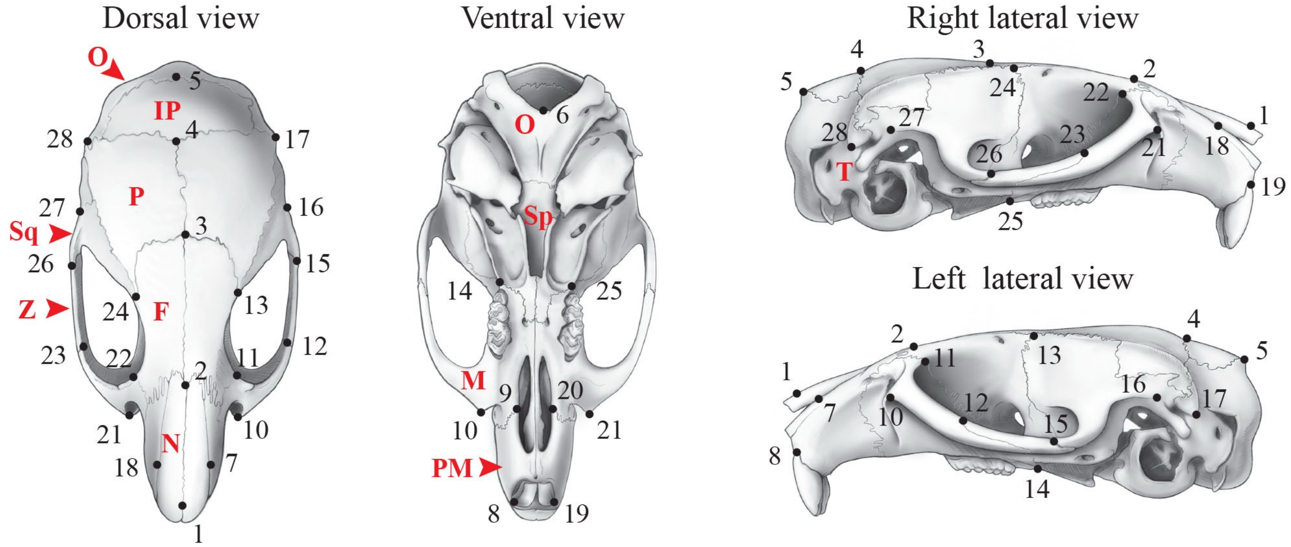

1) Nasal
2) Nasion
3) Bregma
4) Intersection of parietal (P) and interparietal (IP) bones
5) Intersection of the interparietal (IP) and occipital (O) bones at midline
6) Basion
7/18) Anterior-most point at intersection of premaxillae (PM) and nasal (N) bones
8/19) Center of alveolar ridge over maxillary incisor
9/20) Most inferior point on premaxilla-maxilla (PM-M) suture
10/21) Anterior notch on frontal process lateral of infraorbital fissure
11/22) Intersection of frontal process of maxilla (M) with frontal (F) and lacrimal bones
12/23) Intersection of zygomatic process of maxilla with zygoma (jugal, Z)
13/24) Frontal-squamosal (F-Sq) intersection at temporal crest
14/25) Intersection of maxilla (M) and sphenoid (Sp) on inferior alveolar ridge
15/26) Intersection of zygoma (jugal, Z) with zygomatic process of temporal (T), superior aspect
16/27) Joining of squasmosal body to zygomatic process of squasmosal (Sq)
17/28) Intersection of parietal (P), temporal (T) and occipital (O) bones

Supplementary Figure S1

Numbering and description of the landmarks used for 3D geometric morphometric analysis of mouse skulls.
Dorsal, ventral and lateral views of a mouse skull showing the position of the 28 landmarks used for 3D geometric morphometric analysis. The description of each landmark is provided.

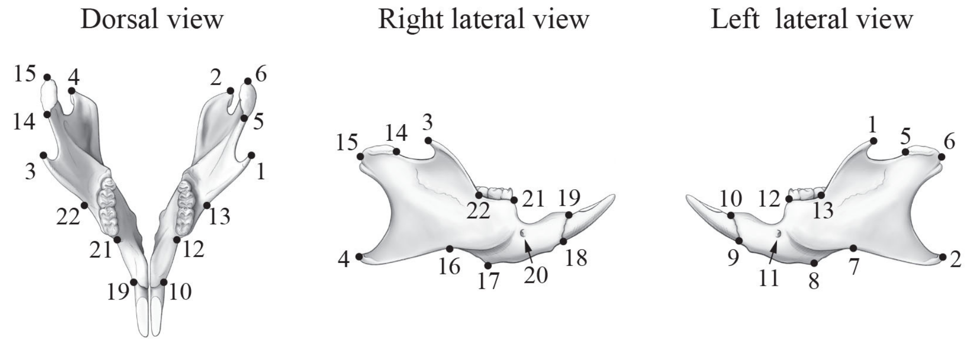

1/3) Coronoid process
2/4) Mandibular angle
5/14) Anterior-most point of mandibular condyle
6/15) Posterior-most point of mandibular condyle
7/16) Superior-most point of inferior border of mandibular ramus (joining of angular notch with corpus)
8/17) Inferior-most point of border of ramus inferior to incisor alveolar
9/18) Inferior-most point on incisor alveolar rim (at bone-tooth junction)
10/19) Superior-most point on incisor alveolar rim (at bone-tooth junction)
11/20) Mandibular foramen
12/21) Anterior point on molar alveolar rim
13/22) Intersection of molar alveolar rim and base of coronoid process

Supplementary Figure S2

Numbering and description of the landmarks used for 3D geometric morphometric analysis of mouse mandible.
Dorsal and lateral views of a mouse mandible showing the position of the 22 landmarks used for 3D geometric morphometric analysis. The description of each landmark is provided.

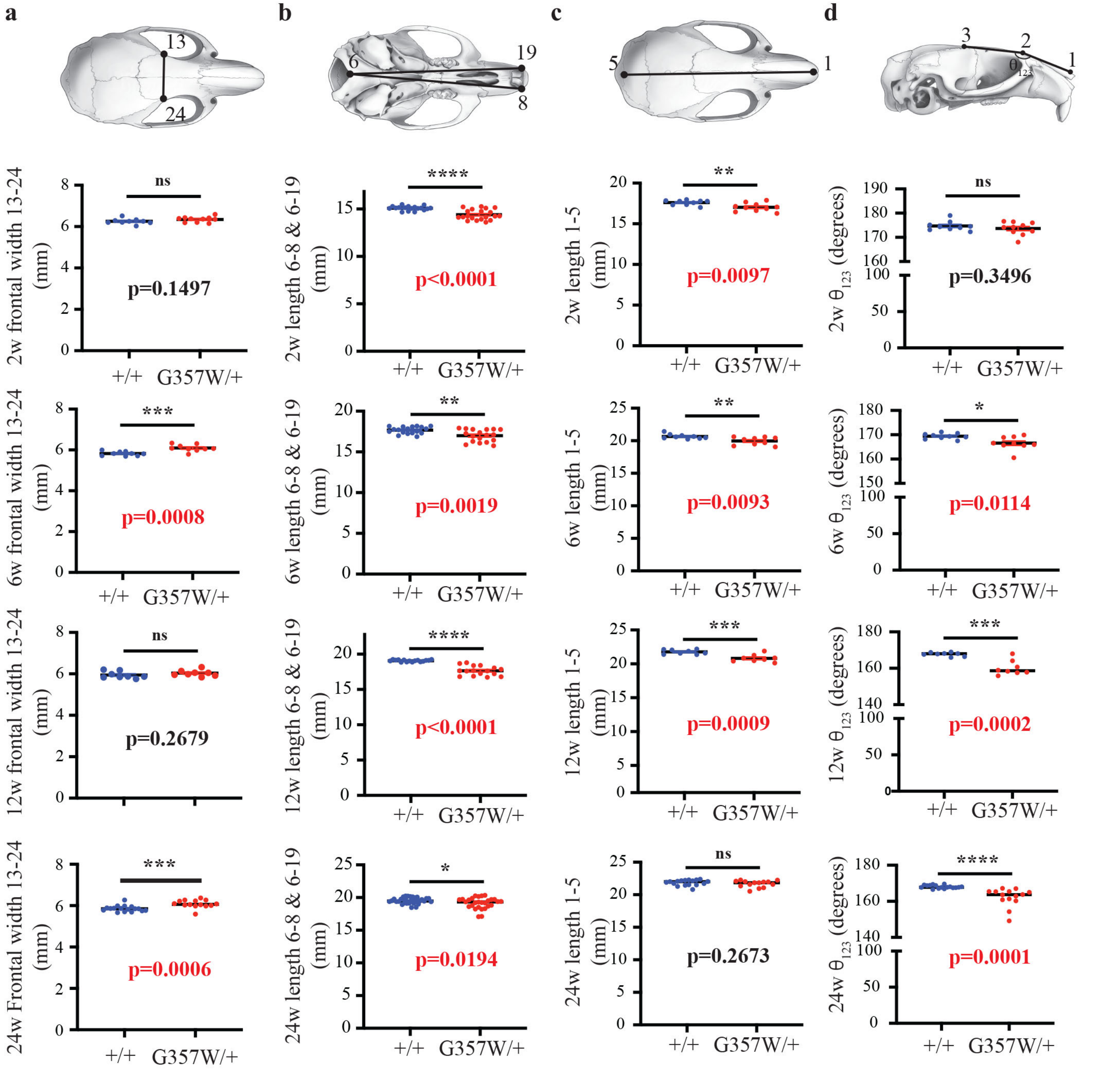

Supplementary Figure S3

Differences in cranial measurement between *Tgfbr2*$^{+/+}$ and *Tgfbr2*$^{G357W/+}$ mice.

(**a**) Measure of the inter-orbital frontal width between the left (landmark #13) and right (landmark #24) frontal-squasmosal intersections at the temporal crest in *Tgfbr2*$^{+/+}$ and *Tgfbr2*$^{G357W/+}$ mice at 2w, 6w, 12w and 24w.

(**b**) Measure of the ventral length of the cranium, between the basion (landmark #6) and the alveolar ridges of the maxillary incisors (landmarks #8 and #19) in *Tgfbr2*$^{+/+}$ and *Tgfbr2*$^{G357W/+}$ mice at 2w, 6w, 12w and 24w.

(**c**) Measure of the dorsal length of the cranium, between the nasion (landmark #1) and the occipital bone (landmark #5) in *Tgfbr2*$^{+/+}$ and *Tgfbr2*$^{G357W/+}$ mice at 2w, 6w, 12w and 24w.

(**d**) Measure of the angle between the nasal and the frontal bones (landmarks #1, #2 and #3) in *Tgfbr2*$^{+/+}$ and *Tgfbr2*$^{G357W/+}$ mice at 2w, 6w, 12w and 24w.

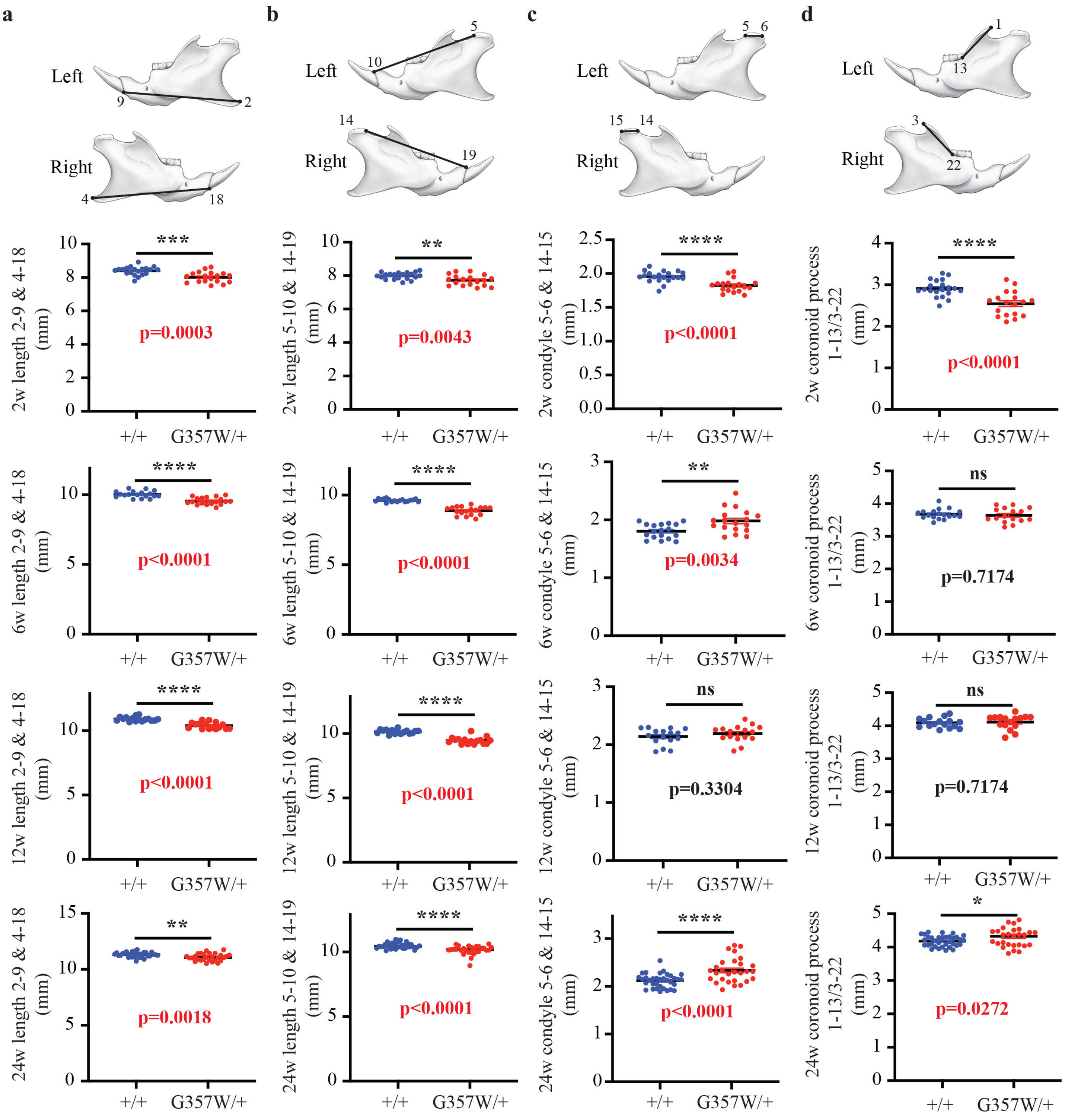

Supplementary Figure S4

Differences in mandible measurement between *Tgfbr2*$^{+/+}$ and *Tgfbr2*$^{G357W/+}$ mice.

(**a**) Measure of the mandible length between the mandibular angle (landmarks #2 and #4) and the incisor alveolar rim (landmarks #9 and #18) in *Tgfbr2*$^{+/+}$ and *Tgfbr2*$^{G357W/+}$ mice at 2w, 6w, 12w and 24w.

(**b**) Measure of the mandible length between the condyle (landmarks #5 and #14) and the incisor alveolar rim (landmarks #10 and #19) in *Tgfbr2*$^{+/+}$ and *Tgfbr2*$^{G357W/+}$ mice at 2w, 6w, 12w and 24w.

(**c**) Measure of the condyle length between landmarks #5 and #6 (left mandible) and between landmarks #14 and #15 (right mandible) in *Tgfbr2*$^{+/+}$ and *Tgfbr2*$^{G357W/+}$ mice at 2w, 6w, 12w and 24w.

(**d**) Measure of the coronoid process length between landmarks #1 and #13 (left mandible) and between landmarks #3 and #22 (right mandible) in *Tgfbr2*$^{+/+}$ and *Tgfbr2*$^{G357W/+}$ mice at 2w, 6w, 12w and 24w.

Statistical significance is indicated using asterisks: *, p<0.05; **, p<0.01; ***, p<0.001; ****, p<0.0001; ns, not significant (p≥ 0.05). p-values are also indicated on the graphs.

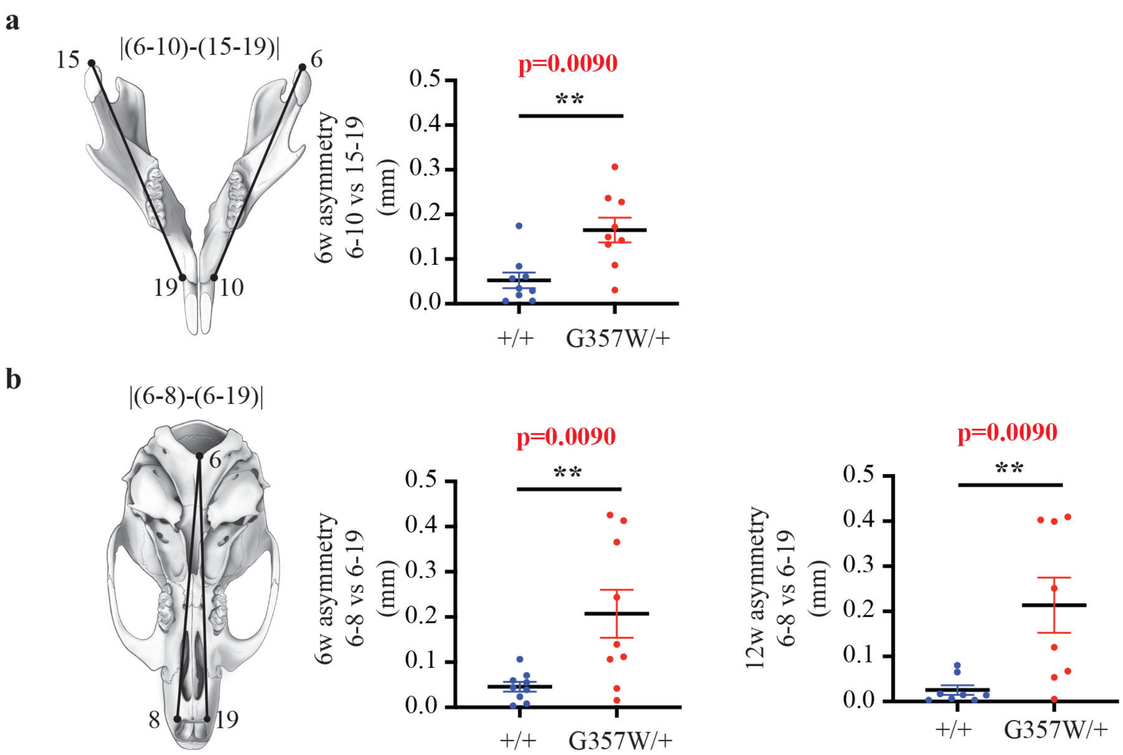

Supplementary Figure S5

Quantification of mandibular and cranial asymmetry in *Tgfbr2*$^{+/+}$ and *Tgfbr2*$^{G357W/+}$ mice.

(**a**) Quantification of the asymmetry between the right and the left mandibles through measurement of the absolute value of the difference in length between the two sides (distance between landmarks #6 and #10 versus distance between landmarks #15 and #19) at 6w.

(**b**) Quantification of the asymmetry between the right and the left sides of the maxilla through measurement of the absolute value of the difference in length between the two sides (distance between landmarks #6 and #8 versus distance between landmarks #6 and #19) at 6w and 12w.

Statistical significance is indicated using asterisks: **, $p<0.01$. p-values are also indicated on the graphs.

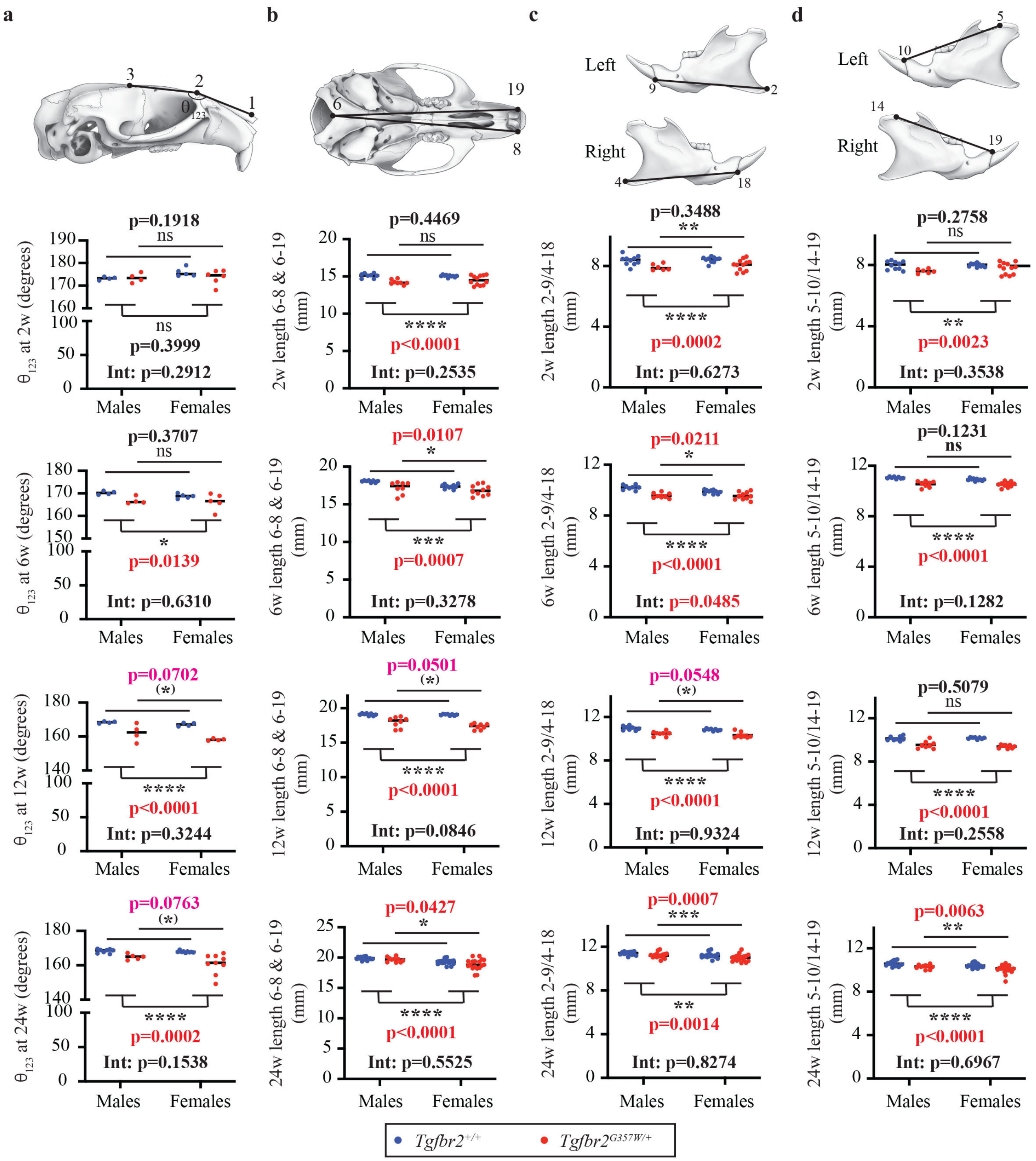

Supplementary Figure S6

Differences in the severity of craniofacial anomalies between males and females in *Tgfbr2$^{G357W/+}$* mice.

(**a**) Two-way ANOVA comparison of the angle between the nasal and frontal bones between males and females *Tgfbr2$^{+/+}$* and *Tgfbr2$^{G357W/+}$* mice at 2w, 6w, 12w and 24w.

(**b**) Two-way ANOVA comparison of the ventral length of the cranium between males and females *Tgfbr2$^{+/+}$* and *Tgfbr2$^{G357W/+}$* mice at 2w, 6w, 12w and 24w.

(**c**) Two-way ANOVA comparison of the mandible length (measured between the mandibular angle (landmarks #2 and #4) and the incisor alveolar rim (landmarks #9 and #18)) between males and females *Tgfbr2$^{+/+}$* and *Tgfbr2$^{G357W/+}$* mice at 2w, 6w, 12w and 24w.

(**d**) Two-way ANOVA comparison of the mandible length (measured between the condyle (landmarks #5 and #14) and the incisor alveolar rim (landmarks #10 and #19)) between males and females *Tgfbr2$^{+/+}$* and *Tgfbr2$^{G357W/+}$* mice at 2w, 6w, 12w and 24w.

Statistical significance is indicated using asterisks: *, $p<0.05$; **, $p<0.01$; ***, $p<0.001$; ****, $p<0.0001$; ns, not significant ($p\geq 0.05$). p-values are also indicated on the graphs (significant in red). p-values that are close to significant (magenta) are annotated as $^{(*)}$. Upper values correspond to sex effect while lower values correspond to genotype effect. The significance of interaction between sex and genotype in indicated at the bottom of each graph (Int).
